# Optimization of Gravity Infusion Protocols for ^177^Lu-DOTATATE Administration in Peptide Receptor Radionuclide Therapy

**DOI:** 10.3390/pharmaceutics18070889

**Published:** 2026-07-20

**Authors:** Salvatore Grasso, Antonio Varallo, Valeria Gaudieri, Michele Klain, Roberta Pastore, Stefania Arena, Caterina Oliviero, Mauro Buono, Daniele Manzi, Regina Schiano, Carmela Nappi, Pasquale Totaro, Rosario Raffaele Bonifacio, Alberto Cuocolo, Stefania Clemente

**Affiliations:** 1 Unit of Medical Physics and Radioprotection, University Hospital Federico II, 80131 Naples, Italy; antonio.varallo@unina.it (A.V.); roberta.pastore@unina.it (R.P.); caterina.oliviero@unina.it (C.O.); mauro.buono@unina.it (M.B.); dan.manzi98@gmail.com (D.M.); reginaschiano.rs@gmail.com (R.S.); stefania.clemente@unina.it (S.C.); 2 Department of Advanced Biomedical Sciences, University of Naples Federico II, 80131 Naples, Italy; valeria.gaudieri@unina.it (V.G.); michele.klain@unina.it (M.K.); cuocolo@unina.it (A.C.); 3 Department of Mental and Physical Health and Preventive Medicine, University of Campania Luigi Vanvitelli, 80138 Naples, Italy; stefania.arena@unicampania.it; 4 IRCCS SYNLAB-SDN, 80143 Naples, Italy; c.nappi@unina.it; 5 Unit of Nuclear Medicine, University Hospital Federico II, 80131 Naples, Italy; pasquale.totaro@unina.it (P.T.); rosario.bonifacio1986@gmail.com (R.R.B.)

**Keywords:** ^177^Lu-DOTATATE, PRRT, infusion kinetics, infusion protocols, fluid dynamics, dose rate monitoring, radiation protection, clinical optimization

## Abstract

**Background:** Peptide receptor radionuclide therapy (PRRT) with ^177^Lu-DOTATATE has become a cornerstone in the management of neuroendocrine tumors (NETs). However, the efficiency of this targeted therapy is highly dependent on the radiopharmaceutical drug delivery system and its infusion kinetics, which influence systemic biodistribution and therapeutic efficacy. This study evaluates various gravity-based infusion fluid dynamics to optimize the drug delivery profile, ensuring standardized delivery while minimizing residual activity. **Methods:** A retrospective analysis of 127 administrations of ^177^Lu-DOTATATE (7.4 GBq per cycle) was conducted across 35 patients with NETs to assess vial dilution kinetics. Four distinct infusion rate (IR) strategies were compared to evaluate mass balance and delivery efficiency: constant infusion rate (1-IR); a single rate increase at 10 min (2-IR); two rate increases at 10 and 30 min (3-IR); and three rate increases at 10, 30, and 40 min (4-IR). **Results:** Stepwise increases in the IR significantly accelerated the vial clearance kinetics and reduced radiopharmaceutical stagnation within the infusion lines. Successful delivery of the target dose (98% of the pre-infusion activity measured in each specific vial) was achieved in 56% of cases with the 1-IR protocol (9 injections) and increased to 87% with 2-IR (45 injections), 97% with 3-IR (60 injections), and 100% with 4-IR (13 injections). **Conclusions:** Modulating fluid dynamics through stepwise IR protocols significantly enhances radiopharmaceutical delivery efficiency. The 3-IR protocol offers a favorable balance between procedural efficiency and clinical safety.

## 1. Introduction

Neuroendocrine tumors (NETs) arise from neuroendocrine cells, primarily within the gastroenteropancreatic (GEP) system [1]. While historically classified as rare malignancies, epidemiological data indicate a steady increase in both incidence and prevalence [1]. This rising trend is largely attributed to advancements in diagnostic imaging modalities, which offer enhanced sensitivity for detecting early-stage disease. Furthermore, the evolution of multimodal therapeutic strategies has significantly improved survival outcomes, particularly for patients with GEP-NETs [2].

A pivotal advancement in managing metastatic or unresectable, well-differentiated GEP-NETs overexpressing somatostatin receptors is peptide receptor radionuclide therapy (PRRT). This approach utilizes the radiolabeled somatostatin analog ^177^Lu-DOTATATE [3], which obtained approval from the European Medicines Agency in 2017 and the Food and Drug Administration in 2018 [4]. ^177^Lu-DOTATATE is a radioligand comprising the somatostatin analog octreotate radiolabeled with ^177^Lu. This isotope boasts ideal theranostic properties: it exhibits β^−^ emission (maximal energy 497 keV) with a mean tissue penetration of 2 mm for targeted cytotoxic action, alongside γ photon emissions (208 keV and 113 keV) that facilitate diagnostic imaging and dosimetric assessment. The efficacy of PRRT relies on the high affinity of radiolabeled peptides for somatostatin receptors, which act as targeting vectors to deliver cytotoxic ionizing radiation directly to tumor cells. This selective mechanism maximizes tumor-absorbed doses while sparing surrounding healthy parenchyma, thereby optimizing the therapeutic index [5]. The standard treatment regimen for eligible patients consists of four cycles of 7.4 GBq administered at 8-week intervals [6]. Throughout the course of therapy, rigorous monitoring of the patient’s clinical and hematological status is mandatory to ensure safety and evaluate treatment response. Prior to ^177^Lu-DOTATATE administration, patients can receive antiemetic prophylaxis to mitigate nausea or vomiting. Concurrently, an intravenous infusion of amino acids (arginine and lysine) reduces tubular reabsorption of the radiopharmaceutical, thereby ensuring nephroprotection. In specific clinical scenarios, long-acting and short-acting somatostatin analogs may also be integrated into the therapeutic regimen. Post-treatment management includes single-photon emission computed tomography/computed tomography imaging to verify tumor targeting and perform accurate dosimetric assessments of organs at risk [7].

While the therapeutic efficacy of PRRT relies on receptor-targeted cytodestruction, the in vivo bioavailability and dose uniformity are critically governed by the drug delivery modality [8,9,10,11]. Maintaining radiation protection standards for both patients and healthcare personnel is paramount during therapeutic administration. Particular vigilance is required regarding the risks of radiopharmaceutical extravasation and environmental contamination; furthermore, standardized intervention protocols must be established to mitigate potential radiation-induced tissue damage [12,13,14]. Gravity-driven infusion remains the most prevalent technique due to its operational simplicity, yet it inherently lacks a universally standardized protocol, leading to substantial variability in formulation delivery efficiency [8]. In a gravity-based setup, the therapeutic vial functions as a dynamic mixing reservoir where the flushing saline continuously dilutes the radiopharmaceutical. This process is governed by complex fluid dynamics, including laminar flow and constant-volume dilution kinetics, which determine the instantaneous concentration profile entering the systemic circulation. Inefficient flow modulation can cause radiopharmaceutical stagnation within the infusion system or sub-optimal clearance due to molecular adhesion of the radiolabeled peptide to the polymer tubing walls. Therefore, analyzing and modulating the infusion rate (IR) kinetics is not merely an operational task, but a fundamental pharmacotechnical requirement to optimize the drug delivery profile. The present study aims to evaluate different stepped gravity-based infusion protocols, focusing on vial clearance kinetics and mass balance optimization, to identify a standardized strategy that ensures maximum therapeutic delivery efficiency.

## 2. Materials and Methods

### 2.1. Patients and ^177^Lu-DOTATATE Administration Protocol

A total of 127 consecutive ^177^Lu-DOTATATE (Lutathera^®^, Novartis, Basel, Switzerland) administrations were retrospectively analyzed from 35 patients (29 men and 6 women) diagnosed with NETs between 2019 and 2025 and enrolled in PRRT. All patients were scheduled to receive four cycles of PRRT (7.4 GBq per cycle) at intervals of 6–8 weeks, according to the practical guidance for radionuclide therapy in NETs [6]. The study was conducted in accordance with the ethical standards laid down in the 1964 Declaration of Helsinki and Good Clinical Practice guidelines.

On the day of therapy, patients were accommodated in a dedicated radiation-shielded room and prepared according to standardized institutional protocols, including the administration of antiemetics and nephroprotective amino acid solutions. The therapeutic vial, containing ^177^Lu-DOTATATE, was initially assayed in the radiopharmacy using a dose calibrator (Gammacal, L’Acn, Lainate, MI, Italy) to determine the pre-administration activity ($A_{V}^{0}$). The vial was subsequently transported to the treatment room, integrated into a gravity-based infusion set, and housed within a lead-shielded cylindrical container to minimize ambient dose rates. For enhanced radiation protection, the assembly was further enclosed in a 1 cm-thick Plexiglas secondary shield. Although a low-Z/high-Z shielding sequence is generally preferred to minimize bremsstrahlung production, this external configuration was adopted as a practical modification to reduce the residual ambient dose rate around the pre-existing lead holder while maintaining a safe working environment.

For technical reproducibility, the gravity-based administration system was assembled as follows. A primary infusion bag containing 0.9% NaCl was suspended from an IV pole and connected through a primary administration line to an inlet spike inserted to the bottom of the ^177^Lu-DOTATATE vial. A second outlet spike was positioned in the vial headspace and connected to the patient’s intravenous line. Controlled saline inflow maintained an approximately constant fluid volume within the vial while continuously diluting the radiopharmaceutical, thereby displacing the diluted solution through the outlet line into the patient’s systemic circulation without the need for a dedicated infusion pump.

#### 2.1.1. Infusion Procedures

The infusion commenced at time *t*_0_ utilizing the gravity-based method over a duration of 30 to 50 min, aimed at maximizing the administered activity. The administration was performed using a standard commercial gravity administration set equipped with medical-grade PVC tubing. Upon completion, the vial was returned to the radiopharmacy hot lab to measure the residual activity ($A_{V}^{R}$). The flow rate of saline (0.9% NaCl), which governs the activity dilution kinetics and the subsequent radiopharmaceutical administration rate, was modulated sequentially between 100 and 250 mL/h. This strategy was designed to ensure a rapid and exhaustive activity transfer into the systemic circulation while mitigating radiopharmaceutical stagnation within the infusion lines, as modeled by Poiseuille-like laminar flow and constant volume dilution kinetics [14].

Patient assignment to a specific infusion protocol followed a pragmatic, non-randomized clinical approach. The initial infusion rate was selected according to the patient’s baseline performance status and overall clinical condition, with more conservative protocols preferred for patients considered clinically frail. During treatment, stepwise increases in the infusion rate were performed only if patients remained clinically stable and did not develop clinically significant infusion-related symptoms, including nausea, a sensation of warmth, or chest tightness. If these symptoms occurred, further rate escalation was withheld and the infusion was continued at the current tolerated rate. Conversely, when clinical tolerance was maintained, progressive flow-rate increments were implemented to enhance dilution of the radiopharmaceutical, limit pressure buildup within the vial, and facilitate complete administration. No predefined hemodynamic thresholds or symptom severity criteria were established; decisions regarding flow-rate escalation were based on the treating physician’s real-time assessment of the patient’s overall clinical stability and tolerability. The infusion strategies were categorized into four distinct protocols based on the frequency and timing of stepwise flow rate escalations:

- Infusion Rate (1-IR, constant rate): A single, steady-state rate of 100 mL/h maintained throughout the entire treatment duration for patients requiring a conservative administration approach.
- 2-Infusion Rate (2-IR, biphasic rate): Features two distinct phases, with an initial rate of 100 mL/h followed by a scheduled rate increase to 150 mL/h implemented at 10 min.
- 3-Infusion Rate (3-IR, triphasic rate): Features three sequential flow rates, starting at 100 mL/h, increasing to 150 mL/h at 10 min, and further escalating to 200 mL/h at 30 min; this protocol was adopted when rate escalation was halted after the second increase due to patient discomfort.
- 4-Infusion Rate (4-IR, tetraphasic rate): The most dynamic approach, involving an initial rate of 100 mL/h and three sequential adjustments to 150 mL/h (at 10 min), 200 mL/h (at 30 min), and 250 mL/h (at 40 min) for patients without clinical contraindications.

The exact infusion rates and timing for each protocol are systematically detailed in Table 1. As shown, flow rates were progressively increased across the steps to maximize the pressure gradient and dilution factor over time, provided the patient showed optimal clinical tolerance.

The implementation of these increments aimed to minimize residual activity in the vial by progressively increasing the pressure gradient and dilution factor within the infusion system. Within the entire study cohort (*n* = 35), longitudinal adherence to a single infusion protocol across all four treatment cycles was observed in 31% of patients. The remaining 69% of patients underwent protocol modifications during their therapeutic course, reflecting a clinical need for dose-rate titration or adaptation to patient tolerance. Specifically, 57%, 9%, and 3% of the cohort received two, three, and four distinct infusion strategies, respectively. This intra-patient variability highlights the adaptive nature of the administration process across the 127 total analyzed therapies.

#### 2.1.2. Real-Time Dosimetric Assessment

**Figure 1:**
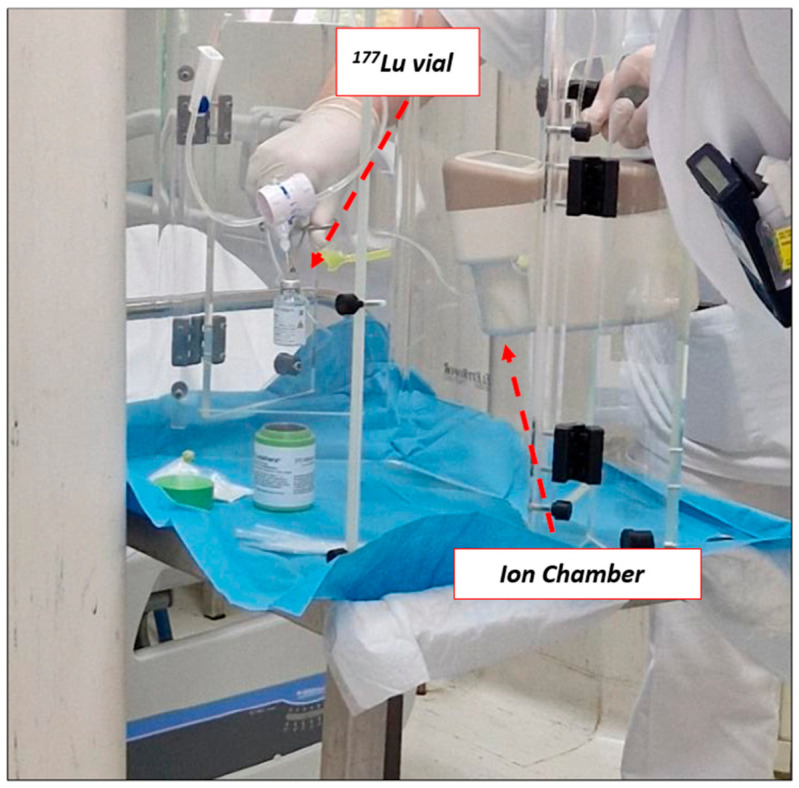
Measurements of ambient dose equivalent rate ${\dot{H}}^{*}\left(10\right)$ using the pressurized ion chamber Ludlum 9DP at 10 cm from the vial.

During each administration, the patient’s clinical and psychophysical status was continuously monitored. Simultaneously, a dedicated medical physics operator performed longitudinal measurements of the ambient dose equivalent rate, ${\dot{H}}^{*}\left(10\right)$, at standardized time intervals (*t_i_* = 0, 5, 10, 20, 30, 45, and 50 min), as illustrated in Figure 1.

These measurements were acquired at a fixed distance of 10 cm from the therapeutic vial using a Ludlum 9DP pressurized ion chamber (PEO B.V., Diegem, Belgium). The device was calibrated for ${\dot{H}}^{*}\left(10\right)$ in μSv/h by the accredited Calibration Laboratory of the Polytechnic University of Milan, utilizing a ^137^Cs source (mean energy: 662 keV; accuracy: 7%). To streamline data acquisition and post-processing, a custom-developed web application was employed, enabling real-time management of medical physics and radiation protection metrics via mobile and desktop interfaces.

#### 2.1.3. Activity Decay and Residual Estimation

The ambient dose equivalent rate at each checkpoint, ${\dot{H}}^{*}$(10, *t_i_*), served as a proxy for estimating the instantaneous residual ^177^Lu activity within the vial. The initial vial activity at the start of the therapy, *A_V_*(*t*_0_), was derived from the pre-administration activity ($A_{V}^{0}$) corrected for physical decay. The instantaneous residual activity within the therapeutic vial, *A_V_*(*t_i_*), was quantified by applying the dose-rate ratio method. This methodology relies on the linear proportionality between the source activity and the measured radiation levels, assuming a constant source-to-detector geometry:

(1) $$A_{V}\left(t_{i}\right)=A_{V}\left(t_{0}\right)\times \frac{{\dot{H}}^{*}({10,t}_{i})}{{\dot{H}}^{*}(10,t_{0})}$$

where ${\dot{H}}^{*}$(10, *t_i_*) and ${\dot{H}}^{*}$(10, *t*_0_) represent the ambient dose equivalent rates measured at the *i*-th checkpoint and at the initiation of the therapy (*t*_0_), respectively. To ensure the robustness of this proxy, the terminal activity calculated at the end of the procedure, *A_V_*(*t_f_*), was systematically cross-validated against the absolute residual activity $A_{V}^{R}$ measured directly via a calibrated dose calibrator. To facilitate real-time clinical workflow, the custom web application automatically computed these instantaneous activity values by entering the raw mSv/h measurements into Equation (1), logging data points at each predefined checkpoint (*t_i_*) to dynamically plot the clearance slopes during the procedure.

#### 2.1.4. Activity Mass Balance and Delivery Efficiency

The net ^177^Lu activity effectively delivered to the patient at any time interval (*t_i_*), denoted as $A_{P}\left(t_{i}\right)$, was derived using the following activity balance equation:

(2) $$A_{P}\left(t_{i}\right)=A_{V}\left(t_{0}\right)-A_{V}\left(t_{i}\right)-A_{L}\left(t_{i}\right)$$

In this formulation, $A_{L}\left(t_{i}\right)$ represents irreversible activity losses from the administration system, including radiopharmaceutical extravasation, leakage from the infusion circuit, or persistent retention within the administration set that is not recovered during saline flushing. Under the operating conditions adopted in this study, no episodes of extravasation or leakage were observed, and continuous saline flushing was implemented to minimize transient adsorption and facilitate recovery of activity retained within the tubing. Therefore, irreversible activity loss was assumed to be negligible ($A_{L}$ ≈ 0) for the purposes of the mathematical model. This approach enabled estimation of the administered activity kinetics while verifying delivery of the prescribed therapeutic activity of 7.4 GBq with high clinical confidence.

#### 2.1.5. Safety Thresholds and Occupational Exposure

**Figure 2:**
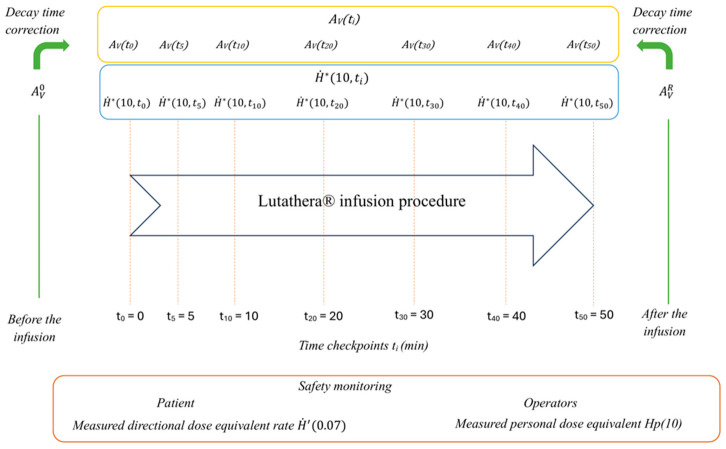
Tracking of dose rate and ^177^Lu activity during the infusion procedure.

To mitigate the risk of radiopharmaceutical extravasation, the directional dose equivalent rate, *H′* (0.07), was periodically monitored at 1 cm from the patient’s skin at the injection site. An operational threshold of 1 mSv/h was adopted as a trigger for suspected extravasation [14]. Furthermore, all personnel adhered to standard operating procedures for emergency radiation protection management [15]. Occupational exposure was quantified using a DMC 3000 electronic dosimeter (Mirion Technologies, Inc., Atlanta, GA, USA), providing real-time *H_p_*(10) values for χ- and γ-radiation. The clinical workflow is schematized in Figure 2.

### 2.2. Assessment of Infusion Protocols and Key Performance Indicators

A comprehensive longitudinal database, comprising procedural and dosimetric data from 127 administrations over a 5-year period, served as the basis for this study. This dataset enabled a multi-parametric statistical analysis to determine the impact of infusion rate (IR) kinetics and total procedure duration on the overall therapeutic delivery. The flow dynamics of the solution were experimentally characterized through real-time dose-rate monitoring. Utilizing the mathematical framework established in Equations (1) and (2), the residual activity in the vial and the corresponding activity delivered to the patient were calculated. Administrations were stratified into four distinct experimental groups (1-IR, 2-IR, 3-IR, and 4-IR) based on the infusion strategy employed. For each cohort, the mean and standard deviation (SD) of the administered activity were quantified at predefined checkpoints, and infusion kinetics curves were generated to model the activity transfer profile.

To objectively evaluate and compare the performance of each protocol, three primary key performance indicators (KPIs) were established:

1. Effectiveness (therapeutic target achievement): Quantified as the total administered activity, representing the system’s capacity to maximize radiopharmaceutical transfer into the systemic circulation. In this study, the “target administered activity” threshold for clinical success was defined as the delivery of ≥98% of the actual pre-infusion activity measured in each specific vial (corresponding to a target mean of approximately 7.25 GBq based on the standard commercial dose).
2. Efficiency (temporal optimization): Defined as the cumulative percentage of administrations achieving the ≥98% actual activity threshold at each temporal checkpoint. This indicator evaluates the protocol’s ability to expedite delivery independently of the absolute starting activity. This approach shortens the period requiring strict patient immobilization during the active administration of the radiopharmaceutical, the phase associated with the highest radiation-safety and procedural demands. Although the concomitant amino acid infusion continues for several hours, reducing the duration of radiopharmaceutical administration substantially decreases patient discomfort and procedural burden. Once radiopharmaceutical delivery has been completed, patients can safely resume limited supervised mobility, such as walking to the restroom while remaining connected to the amino acid infusion via a portable IV pole.
3. Safety (risk and exposure mitigation): Assessed through the frequency of radiopharmaceutical extravasation (*N_e_*) per treatment cycle (*N_e_/N_treatments_*).

Additionally, occupational safety was monitored by quantifying the personal dose equivalent to medical staff, specifically *H_p_*(10) for deep tissue dose.

### 2.3. Statistical Analysis

The comparative analysis of the four infusion protocols was performed by evaluating the statistical distribution of the established key performance indicators. Continuous variables were initially assessed for normality using the Shapiro–Wilk test. Variables with an approximately normal distribution were compared among the four infusion protocols using one-way analysis of variance (ANOVA). When the overall ANOVA was statistically significant, Tukey’s honestly significant difference (HSD) test was applied for post hoc pairwise comparisons while controlling the family-wise error rate. Variables that did not satisfy the assumption of normality were analyzed using the Kruskal–Wallis test. When appropriate, significant Kruskal–Wallis results were further explored by pairwise comparisons using the Mann–Whitney U test with adjustment for multiple testing. Accordingly, the choice of statistical method was determined by the distributional characteristics of each variable. Quantitative results for all primary endpoints are presented as mean ± standard deviation (SD), complemented by 95% confidence intervals (CIs). For all statistical evaluations, a *p*-value < 0.05 was considered the threshold for statistical significance. All analyses were performed using Stata 18.0 (SE—Standard Edition).

## 3. Results

### 3.1. Infusion Procedures

The mean administered activity across the 127 infusion procedures of ^177^Lu-DOTATATE in 35 patients with NETs was 7.37 ± 0.13 GBq, with an average total delivery duration of 45.2 ± 4.7 min. Table 2 reports the number of procedures, the total administered activity, and the calculated total volume of NaCl solution used across the four infusion protocols.

Owing to the different flow-rate protocols, stepwise escalation from the 1-IR to the 4-IR protocol was associated with a progressive increase in the total infused NaCl volume, ranging from a mean of 75.3 ± 9.4 mL to 121.8 ± 10.6 mL. These findings are consistent with the theoretical fluid-dynamic model, whereby increasing the cumulative saline volume through stepwise flow-rate escalation enhances dilution of the radiopharmaceutical within the vial, thereby promoting more efficient vial clearance and reducing residual activity.

**Figure 3:**
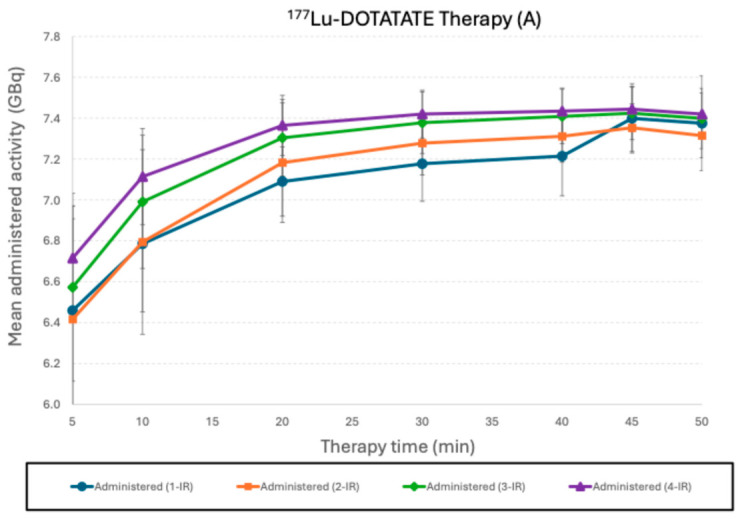

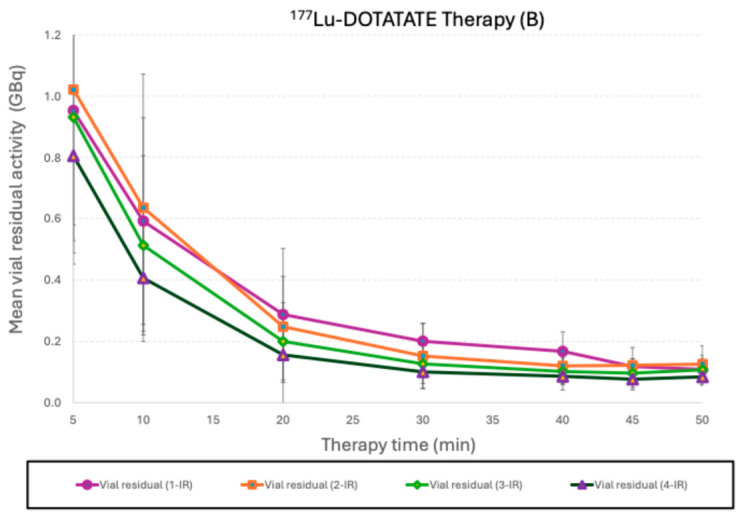
Infusion kinetics profile for the four gravity-based administration protocols as a function of therapy time. (**A**) Mean administered activity successfully delivered to the patient (±SD). (**B**) Mean residual activity remaining in the vial (±SD). As the protocol transitions from 1-IR to 4-IR, the doneresidual activity in the vial decreases more rapidly, resulting in faster activity delivery to the patient.

Consistent with established infusion models, a decreasing trend in the mean residual activity within the vial was observed, accompanied by a reciprocal increase in the cumulative activity delivered to the patient (Figure 3). Progressive escalation from the 1-IR to the 4-IR protocol resulted in increasingly steeper slopes for these kinetics curves. Consequently, the asymptotic values for residual activity were lower for multi-step protocols. Throughout all administrations, the dose rate measured at the patient’s injection site remained between 180 and 200 μSv/h, consistently below the safety alert threshold of 1 mSv/h. No instances of radiopharmaceutical extravasation or environmental contamination were recorded. From a clinical standpoint, the maximum volume administered (~122 mL over 45.2 min) was highly conservative and carried no risk of overload or discomfort for the patient.

### 3.2. Effectiveness

**Figure 4:**
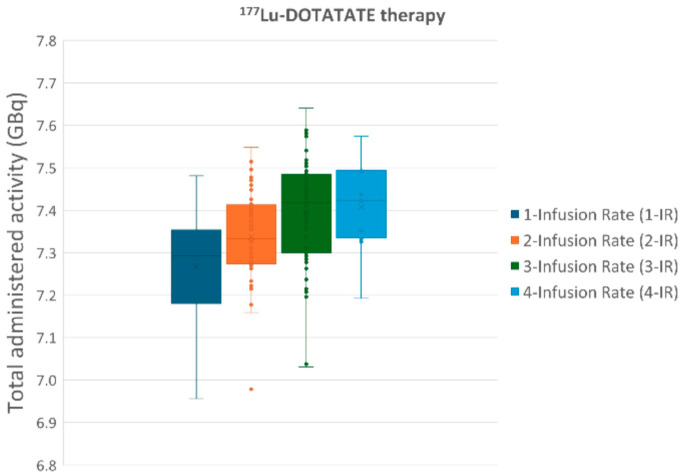
Total administered activity distribution across the four infusion protocols.

The distribution of the total administered activity across the four infusion cohorts was analyzed using non-parametric and parametric modeling (Figure 4). The Mann–Whitney U test (significance level α = 0.05) identified a significant increase in administered activity when comparing the 2-IR to the 3-IR protocol (*p* = 0.001), the 1-IR to the 3-IR protocol (*p* = 0.001), and the 1-IR to the 4-IR protocol (*p* = 0.002). However, the comparison between the 4-IR and 3-IR protocols yielded a *p*-value of 0.82, indicating no significant difference in the total administered activity between these two high-frequency infusion methods.

One-way ANOVA confirmed a significant intergroup variability in total administered activity values across the four protocol groups (*p* < 0.005). Post hoc Tukey’s HSD testing revealed that mean total administered activity values for the 3-IR and 4-IR protocols were significantly higher than those for the 1-IR protocol (both *p* < 0.05). On the contrary, the difference between the 3-IR and 4-IR protocols remained non-significant (*p* = 0.98).

### 3.3. Efficiency and Safety Indicators

**Figure 5:**
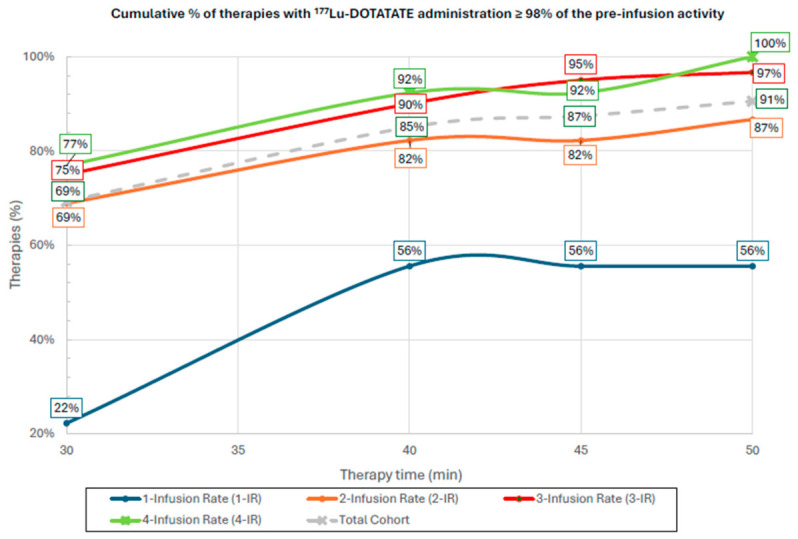
Cumulative percentage of therapies achieving a total administered activity ≥ 98% of the pre-infusion activity (the “target administered activity”) across the treatment checkpoints.

Efficiency was assessed by calculating the cumulative percentage of treatments achieving the minimum total administered activity threshold of ≥98% of the actual pre-infusion activity (the “target administered activity”) at each checkpoint. The 3-IR and 4-IR protocols demonstrated superior performance, reaching the target in 97% and 100% of infusions, respectively. For both strategies, an achievement rate of over 90% was maintained from 40 min onward (Figure 5).

The total number of extravasation events observed throughout all 127 administrations was zero (*N_e_* = 0). Professional radiation exposure for the nursing staff and medical physicists was monitored (Table 3).

## 4. Discussion

Gravity-based infusion remains the foundational modality for the administration of ^177^Lu-DOTATATE in patients undergoing PRRT [8]. Within the framework of clinical quality assurance and procedural optimization involving ionizing radiation, this study evaluated the impact of various infusion strategies. Our findings demonstrate that multi-step protocols (3-IR and 4-IR) exhibit superior delivery efficiency compared to constant or single-increment strategies (1-IR and 2-IR), with the performance gap in total administered activity reaching high statistical significance.

From a fluid dynamics perspective, these results are elucidated by the dilution kinetics within the infusion system. The therapeutic vial acts as a mixing reservoir where the saline solution continuously dilutes the radiopharmaceutical. The modulation of the flow rate directly accelerates this process, leading to a faster peak concentration and a more exhaustive transfer of the 7.4 GBq nominal activity. Consequently, higher flow rates optimize the clinical workflow, thereby minimizing patient discomfort and reducing occupational radiation exposure for healthcare personnel.

The progressive escalation of the flow rate (starting at a conservative level and increasing as the activity concentration in the vial decreases) serves a critical dual purpose: while in the initial phase a moderate starting rate enhances patient tolerability, allowing for the effective management of acute, infusion-related side effects such as nausea, in the subsequent phases the incremental flow increases ensure the complete displacement of the radiopharmaceutical, preventing activity stagnation within the vial or infusion lines due to gravitational settling or molecular adhesion to the polymer tubing walls.

Notably, although the 4-IR protocol achieved the target activity in all cases, the statistical analysis indicates no significant incremental benefit over the 3-IR strategy. From a clinical optimization perspective, the 3-IR approach represents the efficiency plateau for standard procedures. By achieving nearly identical therapeutic effectiveness with fewer manual rate adjustments, the 3-IR protocol (150 mL/h for 0–10 min, 200 mL/h for 10–30 min, and 250 mL/h for 30–45 min) streamlines the clinical workflow and adheres to the ALARA principle without compromising treatment quality.

Prior studies demonstrated that ^177^Lu-DOTATATE therapy is safe and effective in controlling the burden of disease in patients with GEP-NETs [3,16]. Nevertheless, clinical judgment remains paramount when selecting the infusion strategy. The 1-IR and 2-IR protocols remain important options for clinically vulnerable patients who may not tolerate higher infusion volumes or progressive flow-rate escalation. Furthermore, careful monitoring of both the patient and the infusion system is essential throughout radiopharmaceutical administration. Although higher flow rates may theoretically increase hydrostatic pressure within the infusion circuit and at the vial connections, potentially increasing the risk of micro-leakage or mechanical extravasation, this risk can be minimized by meticulous assembly of the administration system, verification of all connections before treatment, gradual rather than abrupt flow-rate escalation, and continuous supervision by trained personnel. Progression to each subsequent flow-rate level was based on the patient’s real-time clinical tolerance and was discontinued whenever infusion-related symptoms or technical concerns arose. Under these operating conditions, no episodes of radiopharmaceutical extravasation or leakage were observed in our cohort, supporting the feasibility and safety of the proposed stepped-rate approach, while acknowledging that gravity-based administration systems are not inherently free of technical limitations. Dosimetric data further confirmed that the annual occupational radiation exposure of all personnel remained well within the limits recommended by the International Commission on Radiological Protection (ICRP) and current national legislation [17,18,19].

Despite the encouraging outcomes, several limitations warrant consideration. First, the retrospective, single-center design, with a small number of patients may limit the generalizability of the findings. A larger cohort would enhance the statistical power and robustness of our conclusions. Second, the non-randomized, pragmatic allocation of patients to infusion strategies introduces a potential selection bias, as patients with more favorable baseline clinical conditions were more likely to be assigned to the accelerated 3-IR or 4-IR protocols. This pragmatic allocation also resulted in unequal group sizes, reflecting routine clinical practice rather than balanced experimental group assignment. Consequently, the smaller cohorts may have provided lower statistical power and less precise estimates for between-group comparisons. Therefore, the observed differences among infusion protocols should be interpreted with appropriate caution and considered hypothesis-generating until confirmed in larger prospective studies with more balanced group distributions. Third, while occupational exposure and extravasation events were meticulously monitored, the study lacked standardized patient-reported outcome measures to objectively quantify subjective discomfort or long-term safety perceptions. Our findings are specific to the vial geometry and gravity-driven sets employed at our institution; therefore, the absolute flow rates and timing may require local calibration and validation in different clinical environments. An additional limitation of this study is that several patients contributed more than one treatment administration. Although each administration represented a distinct clinical event and the infusion protocol was selected independently for each cycle based on the patient’s contemporaneous clinical status, infusion tolerability, and technical considerations, repeated administrations from the same patient cannot be assumed to be statistically independent. Consequently, the use of conventional between-group statistical tests may not fully account for within-patient correlation. These findings should therefore be interpreted with appropriate caution and warrant confirmation in larger prospective studies employing repeated-measures statistical models. Due to the non-randomized protocol allocation and single-center framework, selection bias cannot be ruled out. Consequently, our findings should be interpreted as an exploratory proof-of-concept rather than a definitive justification for a new universal standard.

## 5. Conclusions

This study demonstrates that while gravity-based infusion is a safe and reliable method for ^177^Lu-DOTATATE administration, its overall efficiency is significantly dictated by patient-specific optimization of the infusion rate. Our findings suggest that the 3-IR protocol offers a favorable balance between procedural efficiency and clinical safety, and may represent a reasonable option for institutions seeking to streamline administration; however, larger, prospective studies are needed before it can be recommended as a standard of care. While reducing total administration time to approximately 45 min (or less) is desirable to alleviate patient distress and minimize occupational radiation exposure, it is imperative that such accelerated durations do not compromise the quantitative transfer of the radiopharmaceutical activity. Ultimately, a dynamic and personalized approach to flow-rate adjustments, supported by real-time dose-rate monitoring, ensures high-quality therapeutic delivery while maintaining rigorous radiation protection standards.

## Figures and Tables

**Table 1:** Operational parameters of the four gravity-based infusion protocols. The table details the specific saline flow rates (mL/h) and chronological phases (*t*_0_ to end of infusion) configured for each administration strategy based on patient tolerance and protocol design.

Infusion Protocol	Phase 1 (*t*_0_ to 10 min)	Phase 2 (10 to 30 min)	Phase 3 (30 to 40 min)	Phase 4 (40 min to End)
1-IR (Constant)	100	100	100	100
2-IR (Biphasic)	100	150	150	150
3-IR (Triphasic)	100	150	200	200
4-IR (Tetraphasic)	100	150	200	250

**Table 2:** Total administered activity in the four infusion protocols.

Infusion Protocol	Administration (*n*, %)	Total Administered Activity (GBq)	Total NaCl Volume Infused (mL)
1-IR	9 (7%)	7.26 ± 0.14	75.3 ± 9.4
2-IR	45 (36%)	7.34 ± 0.11	104.7 ± 12.6
3-IR	60 (47%)	7.40 ± 0.13	117.3 ± 13.8
4-IR	13 (10%)	7.41 ± 0.10	121.8 ± 10.6

Data are presented as *n* (%) or mean ± SD.

**Table 3:** Occupational exposure of nurses and medical physicists performing ^177^Lu-DOTATATE PRRT in the Nuclear Medicine Unit at University Hospital Federico II of Naples.

Professional	Number	Mean Dose (μSv)
Nurse	5	1.34 ± 0.6
Medical Physicist	2	1.21 ± 0.7

Data are presented as mean ± SD.

## Data Availability

The data presented in this study are available on request from the corresponding author. The data are not publicly available due to privacy restrictions.

## Abbreviations

The following abbreviations are used in this manuscript:

NETs	Neuroendocrine tumors
GEP	Gastroenteropancreatic
PRRT	Peptide receptor radionuclide therapy
IR	Infusion rate
ANOVA	Analysis of variance
